# Incretins and SGLT-2i Therapy of Type 2 Diabetes – Real Life Study of Their Therapeutic and Economic Effects

**DOI:** 10.3389/fphar.2019.00364

**Published:** 2019-04-16

**Authors:** Zornitsa Mitkova, Konstantin Mitov, Vasil Valov, Manoela Manova, Alexandra Savova, Maria Kamusheva, Dimitar Tcharaktchiev, Zhivko Angelov, Galia Angelova, Guenka Petrova

**Affiliations:** ^1^Faculty of Pharmacy, Medical University of Sofia, Sofia, Bulgaria; ^2^Department of Endocrinology, University Specialized Hospital for Active Treatment of Endocrinology, Sofia, Bulgaria; ^3^Adiss Lab Ltd., Sofia, Bulgaria; ^4^Institute of Information and Communication Technologies, Bulgarian Academy of Sciences, Sofia, Bulgaria

**Keywords:** incretins, SGLT-inhibitors, diabetic incidents, HbA1c level, cost study

## Abstract

**Aim:**

Incretins [dipeptidyl peptidase-4 inhibitors (DPP-4i) and glucagon-like peptide 1 RA (GLP-1 RA)] and sodium-glucose cotransporter-2 inhibitors (SGLT-2i) groups are now routinely used for type 2 diabetes therapy and comprise a large number of medicinal products. The long term therapeutic and economic effect of the incretins’ and SGLT-2i in real life setting is not well documented. The goal of the current study is to analyze the cost and results of incretins and SGLT-2i based therapy for type 2 diabetes in Bulgaria.

**Methods:**

The study uses information about the changes in glycated hemoglobin (HbA1c) level from the National diabetes register for 6122 patients and cost paid by the National Health Insurance Fund (NHIF) for diabetes complications, and medicine prices.

**Results:**

The results show that after the therapy patients achieved excellent diabetes control. There were no HbA1c values less than 6% before treatment. After the therapy, 3356 people showed values less than 7% HbA1c. It is considered very good diabetic control. The number of people with HbA1c above 8% is decreasing significantly. The number of people with values above 9% is decreasing by almost four times. HbA1c level decreases with the highest percentage for the patients treated with GLP-1 RA, followed by those treated with DPP-4i and SGLT-2i. For a year NHIF reimbursed 5.25 million BGN for incretins and SGLT-2i therapy. NHIF can save between 306 and 510 thousand BGN from incidents that have not occurred as a result of 5 years of therapy.

**Conclusion:**

Incretins [dipeptidyl peptidase-4 inhibitors (DPP-4i) and glucagon-like peptide 1 receptor agonists (GLP-1 RA)] and sodium-glucose linked transporter-2 inhibitors (SGLT-2i) therapy steadily decreases the HbA1c level, and risk of developing diabetic incidents is reduced to between 333 and 465 cases among 6122 treated patients. Avoided cost for therapy of diabetes incidents account for between 305 and 510 thousand BGN.

## Introduction

Incretin (DPP-4i and GLP-1 RA) and SGLT-2i groups are now routinely used for type 2 diabetes therapy and comprise a large number of medicinal products (Cheung et al., 2009; Lovshin and Drucker, 2009; Drucker et al., 2010). They may be used as a second or third line medication for people with type 2 diabetes after prescribing MET and sulphonylureas, and as an alternative to thiazolidinedione medication (American Diabetes Association, 2016). DPP-4i are a new, developing group, a regulator of incretin hormones, prolonging the incretin’s effects (Anitha et al., 2013; Röhrborn et al., 2015). GLP-1 receptor agonists or incretin mimetics are agonists of the GLP-1 receptor (Freeman and Unger, 2008). SGLT-2i inhibitors have been approved for type 2 diabetes therapy since 2013 and are a new group of oral medications, acting by helping the kidneys to lower blood glucose levels (Nauck, 2013).

As relatively new medicines for diabetes therapy, their efficacy, safety, and cost-effectiveness are intensively studied all over the world (Gachev, 2004; Ross and Ekoe, 2010; Butler et al., 2013). The long term therapeutic and economic effect of the incretins and SGLT-2i in real life setting is not well documented, especially in Bulgaria, which stimulates our study (Cernea, 2011; Pérez et al., 2015).

The goal of the current study is to analyze the cost and results of incretins and SGLT-2i based therapy for type 2 diabetes in Bulgaria. The study observes variations in HbA1c level; calculate the expected risk of diabetes complications appearance and their cost.

The point of view is that of the payer NHIF and the time horizon is 5 years.

## Materials and Methods

This is an observational study based on the officially reported results for diabetic population therapy in which incretin and SGLT-2i base therapy achieved decrease in HbA1c level over a 1 year period. The observation includes all patients treated during the period 2012–2016 after the introduction of the therapeutic groups in the practice.

The changes in HbA1c levels are extracted from the records of 705,515 type 2 diabetic patients, summarized in the National diabetes register. The selected patients are treated with medicines belonging to DPP-4i (sitagliptin, vildagliptin, saxagliptin, and linagliptin), SGLT-2i (dapagliflozin and empagliflozin), GLP-1 RA (exenatide, liraglutide, lixisenatide, and exenatide extended-release), DPP-4i + MET (sitagliptin/MET; vildagliptin/MET, saxagliptin/MET, and linagliptin/MET), and SGLT-2i + MET (dapagliflozin/MET and empagliflozin/MET). Information about the initial and final HbA1c values is found for 10,547 patients. Patients have been separated according to the therapeutic group of medicines, and recorded changes in HbA1c. Out of 10,547 patients 6122 had a decrease in the HbA1c level and they were included in this analysis.

Changes in HbA1c levels has been used as the main criterion for the outcome of therapy. According to NHIF rules the patients that did not have a decrease in HbA1c level after being transferred to incretin and SGLT-2i therapy should stop the therapy and, therefore, they were excluded from the study. The average decrease in HbA1c was calculated for the whole observed 5-year period.

Literature evidence confirming a decrease in the RR and number of diabetic incidents of about 1% decrease in HbA1c are included in the study (Siratton et al., 2000; Gale, 2014; Table 1). RR is a ratio of the probability of diabetic incidents appearing in the group with decrease in HbA1c level and those with no decrease.

**Table 1:** Decrease in the RR and number of diabetic incidents with 1% decrease in HbA1c (Siratton et al., 2000; Gale, 2014).

Diabetic incidents	RR decrease for 1% decrease in HbA1 (95% CI)	Expected number of incidents for 1000 persons per different HbA1c levels					
		<6	6 to <7	7 to <8	8 to <9	9 to <10	>10
Any end point related to diabetes	21 (17–24)	35.9	48.7	65.5	74.5	62.5	65.9
Death related to diabetes	21 (15–27)	8.9	12.0	19.9	23.5	29.5	33.0
All cause mortality	14	17.0	23.3	30.0	31.8	37.0	40.7
Fatal and non-fatal myocardial infarction	14 (8–21)	16.0	20.8	29.2	30.0	39.6	38.6
Fatal and non-fatal stroke	12	4.3	6.6	8.3	7.4	6.7	12.0
Microvascular end points	37 (33–41)	6.1	9.3	14.2	22.8	40.4	57.8
Cataract extraction	19	4.1	4.5	4.9	6.9	6.6	14.4
Amputation or death from peripheral vascular disease	43	1.2	1.2	2.6	4.0	10.9	12.2
Heart failure	16	2.3	3.4	5.0	4.4	5.0	11.9

Because of the widely accepted evidence of the cited literature about the influence of the decrease in HbA1c level on the RR reduction, we assume that the same RR reduction RR will apply in our cohort. The decrease in the RR among the observed population is calculated by multiplying the average HbA1c decrease by the treated subjects with the RR from Table 1. This calculation is performed for 6122 subjects.

The average follow-up period for the changes in the RR of diabetes complication is varies between 10.4 years for all-cause mortality and 10 years for other incidents (Gale, 2014). Therefore, we assumed a 10 year follow up period.

We calculated 3 types of direct medical costs: the cost of medicines; hospitalizations in case of complications, by assuming one incident per life time per patient; and the yearly ambulatory cost of each incident.

The cost paid by the NHIF for incretins and SGLT-2i therapy was calculated by multiplying the average reimbursed cost for each group with the number of treated patents and then recalculated for 5 years (National Health Insurance Fund, 2018).

The cost of avoided incidents was calculated by multiplying their number with the cost of one hospital incident per event, per patient life time, and the cost for 10 years ambulatory therapy – Table 2. Cost for hospital therapy is taken from the NHIF tariff and the cost for ambulatory therapy from the literature (Ministry of Health, 2016a; Ostrowska et al., 2017).

**Table 2:** Hospital and ambulatory cost of health care services.

Incident	NHIF tariff cost [BGN]	NHIF clinical path tariff	Yearly ambulatory cost of therapy [BGN; 17]
Any end point related to diabetes	600	No104	427.92
Death related to diabetes	600	No104	0
All cause mortality	2134	No129	0
Fatal and non-fatal myocardial infarction^∗^	200	No47.1; 47.2	51.22
Fatal and non-fatal stroke^∗^	650.56	No1; 2; 3; 4	43.13
Microvascular end points^∗∗^	744	No56; 62; 69; 129; 133;138	36.38
Cataract extraction	360	No131	10.08
Amputation or death from peripheral vascular disease	2050	No215	20.68
Heart failure	420	No52	112.72

*^∗^Weighted average cost derived though multiplying the expected number of patients with the unit tariff cost. ^∗∗^Average tariff cost for related incidents.*

Not all risks for diabetic incidents had tariff cost and, therefore, several assumptions have been made in Table 2. The first one is that, before the death related to diabetes, patients were hospitalized for diabetic incident. The second is that patients with fatal and non-fatal strokes will follow the expected in the tariff numbers and cost of incidents. Microvascular incidents, namely retinopathy; nephropathy and neuropathy were calculated as average from all corresponding tariff costs due to their clinical diversity. In the case of death from any reason it was assumed that patients were hospitalized for emergency non-trauma therapy. Ambulatory therapy costs were calculated for 10 years corresponding to an average follow-up period in UKPDS 35 and then recalculated for 5 years.

All costs are presented in national currency (BGL) at the exchange rate of 1 BGN = 0.94 Euro.

Descriptive statistics and *t*-test analysis were performed to evaluate the confidence interval and differences in the risk reduction.

## Results

### Medical and Social Consequences

**FIGURE 1:**
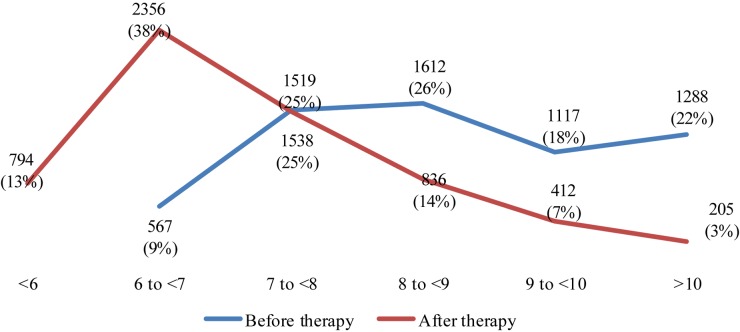
Changes in number patients per HbA1c level before and after the therapy with incretins and SGLT-2i.

Figure 1 presents the changes in average HbA1c level before and after the therapy with incretins and SGLT-2i. There were no HbA1c values less than 6% before the therapy, and prevailing part of patients were above 9% HbA1c. After the therapy, 3356 people showed values less than 7% HbA1c. It is considered very good diabetic control. The number of people with HbA1c above 8% is decreasing significantly. The number of people with values above 9% is decreasing almost 4 times as much (617 vs. 2415 with values above 9%, after and before the therapy, respectively). The red line that is performing the changes in HbA1c after therapy is moving to lower levels for most of the patients – Figure 1.

Glycated hemoglobin level decreases the highest percentage for the patients treated with GLP-1 RA (1.76%), followed by those treated with DPP-4i (1.43 for monotherapy and 1.71 for combination therapy with MET), and SGLT-2i – Table 3. The one-way *t*-test was applied to check whether the reduction in HbA1c level is strongly negative for all patients receiving a particular therapeutic group. All reductions in HbA1c are statistically significant negative at the level of significance 0.05. DPP-4i was first introduced in the reimbursement system, and the last one was the SGLT-2i products.

**Table 3:** Reduction in HbA1c according to therapeutic group.

HbA1c decrease in %	DPP-4i	DPP-4i + MET	GLP-1 RA	SGLT-2i	SGLT-2i + MET	Total average reduction
Average decrease for 6122 patients with positive clinical result **(***p*-value**)**	−1.43 (*p* < 0.001)	−1.71 (*p* < 0.001)	−1.76 (*p* < 0.001)	−1.71 (*p* = 0.002)	−1.46 (*p* = 0.001)	−1.67 (*p* < 0.001)

Patients on SGLT-2i as monotherapy (*n* = 155) or combination with MET (*n* = 86) are relatively small number due to later introduction of products from both therapeutic group in the reimbursement – Table 4.

**Table 4:** Glycated hemoglobin level per therapeutic groups in the beginning and end of the observed period (%).

In the beginning	<6%	6–7%	7–8%	8–9%	9–10%	>10%	Total per group
DPP-4i		114 (20.1%)	263 (17.1%)	264 (16.4%)	165 (14.8%)	141 (10.9%)	**947**
DPP-4i + MET		351 (61.9%)	900 (58.5%)	916 (56.8%)	683 (61.2%)	866 (67.2%)	**3716**
GLP-1 RA		83 (14.6%)	303 (19.7%)	373 (23.1%)	229 (20.5%)	230 (17.9%)	**1218**
SGLT-2i		10 (1.8%)	44 (2.9%)	37 (2.3%)	29 (2.6%)	35 (2.7%)	**155**
SGLT-2i + MET		9 (1.6%)	28 (1.8%)	22 (1.4%)	11 (0.9%)	16 (1.2%)	**86**
Total per HbA1c level		567	1538	1612	1117	1288	6122
**At the end of the period**							
DPP-4i	117 (14.7%)	394 (16.7%)	242 (15.9%)	115 (13.8%)	48 (11.7%)	31 (15.1%)	**947**
DPP-4i + MET	484 (60.9%)	1365 (57.9%)	939 (61.8%)	517 (61.8%)	272 (66%)	139 (67.8%)	**3716**
GLP-1 RA	165 (20.8%)	505 (21.4%)	276 (18.2%)	163 (19.5%)	77 (18.7%)	32 (15.6%)	**1218**
SGLT-2i	16 (2%)	59 (2.5%)	42 (2.8%)	26 (3.1%)	10 (2.4%)	2 (0.9%)	**155**
SGLT-2i + MET	12 (1.5%)	33 (1.4%)	20 (1.3%)	15 (1.8%)	5 (1.2%)	1 (0.5%)	**86**
Total per HbA1c level	794	2356	1519	836	412	205	6122

**FIGURE 2:**
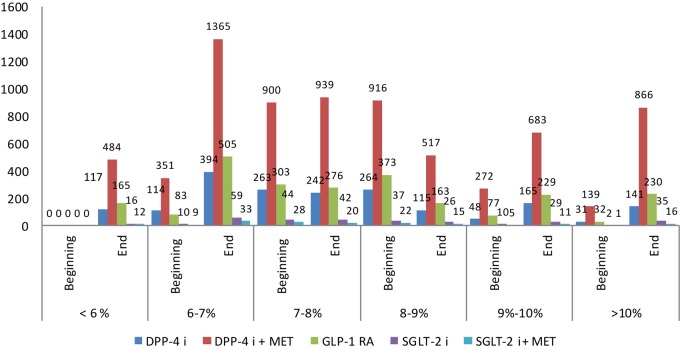
Glycated hemoglobin (HbA1c) at the beginning and end of observation per therapeutic groups.

Graphical presentation of the HbA1c values per therapeutic group shows that more patients on DPP4i + MET and GLP-1 RA decrease their HbA1c below 7–8% (Figure 2).

Reduction in HbA1c level about 1% leads to lower diabetic incidents. The reduction is between 20 for fatal and non-fatal strokes to 62 for microvascular complications (Table 5). All total average risk reductions are higher than zero in 95% CI and, therefore, are statistically significant.

**Table 5:** Reduction in diabetic incidents.

	DPP-4i	DPP-4i + MET	GLP-1 RA	SGLT-2i	SGLT-2i + MET	Total average RR reduction per incident (95% CI)
Any end point related to diabetes	30	36	37	36	31	35 (30–38)
Death related to diabetes	30	36	37	36	31	35 (30–38)
All cause mortality	20	24	25	24	20	23 (20–26)
Fatal and non-fatal myocardial infarction	20	24	25	24	20	23 (20–26)
Fatal and non-fatal stroke	17	21	21	21	18	20 (17–22)
Microvascular end points	53	63	65	63	54	60 (53–67)
Cataract extraction	27	32	33	32	28	31 (27–34)
Amputation or death from peripheral vascular disease	61	74	76	74	63	70 (61–79)
Heart failure	23	27	28	27	23	26 (23–29)
Total average RR reduction per therapeutic group	282	337	347	337	288	329

The patients treated with GLP-1 RA and those on DPP4i + MET show the highest total average RR reduction. Reduction of incidents depends on the HBA1c level reduction between the beginning and end of observation Table 5.

In the beginning of the therapy the expected number of diabetic incidents is 1316 depending on the results from the initial values of HbA1c, where the highest is the risk for any end point related diabetes incidents (*n* = 403), followed by all-cause mortality – Table 6. No patient with HbA1c values less than 6% were observed in the beginning and, therefore, the number of incidents in this group is zero.

**Table 6:** Expected number of incidents before the treatment.

Diabetic incidents	HbA1c level in %						
	<6	6 to <7	7 to <8	8 to <9	9 to <10	>10	Total number of expected incidents for all patients before the therapy
Any end point related to diabetes	0.0	27.6	100.7	120.1	69.8	84.9	403
Death related to diabetes	0.0	6.8	30.6	37.9	33.0	42.5	151
All cause mortality	0.0	13.2	46.1	51.3	41.3	52.4	204
Fatal and non-fatal myocardial infarction	0.0	11.8	44.9	48.4	44.2	49.7	199
Fatal and non-fatal stroke	0.0	3.7	12.8	11.9	7.5	15.5	51
Microvascular end points	0.0	5.3	21.8	36.8	45.1	74.4	183
Cataract extraction	0.0	2.6	7.5	11.1	7.4	18.5	47
Amputation or death from peripheral vascular disease	0.0	0.7	4.0	6.4	12.2	15.7	39
Heart failure	0.0	1.9	7.7	7.1	5.6	15.3	38
Total number of expected incidents	**1315**

During the therapy with incretins and SGLT-2i for the period 2012–2016 the diabetes control is changing and the number of people with better control is increasing. This leads to the decrease in the number of expected incidents to 983 (Table 7). The total number of all diabetes related incidents is reduced to 344, whereas death due to diabetes decreased to 104 people. A death due to any other reason decreased to 164 people. The avoided cases, due to different diabetic complications or fatal conditions and incidents, number 333. Most of the changes in the expected incidents were found to be statistically significant except the fatal and non-fatal strokes, cataract extraction, and heart failure, probably due to a small number of affected people.

**Table 7:** Expected number of incidents after the treatment.

Diabetes incidents	Hba1c						Total number of expected incidents after the therapy	Difference in incidents number “before and after the therapy
	<6	6 to <7	7 to <8	8 to <9	9 to <10	>10		
Any end point related to diabetes	28.5	114.7	99.5	62.3	25.8	13.5	344	59 (*p* = 0.036)
Death related to diabetes	7.1	28.3	30.2	19.6	12.2	6.8	104	47 (*p* = 0.004)
All cause mortality	13.5	54.9	45.6	26.6	15.2	8.3	164	40 (*p* = 0.039)
Fatal and non-fatal myocardial infarction	12.7	49.0	44.4	25.1	16.3	7.9	155	44 (*p* = 0.021)
Fatal and non-fatal stroke	3.4	15.5	12.6	6.2	2.8	2.5	43	8 (*p* = 0.411)
Microvascular end points	4.8	21.9	21.6	19.1	16.6	11.8	96	88 (*p* < 0.001)
Cataract extraction	3.3	10.6	7.4	5.8	2.7	3.0	33	14 (*p* = 0.118)
Amputation or death from peripheral vascular disease	1.0	2.8	3.9	3.3	4.5	2.5	18	21 (*p* = 0.020)
Heart failure	1.8	8.0	7.6	3.7	2.1	2.4	26	12 (*p* = 0.134)
Total/difference							983	333

Similar calculations could also be performed with average RR values without separating incidents to level of HbA1c achieved – Table 8. This calculation is estimating the avoided diabetic incidents to 458 cases on average. All averages of avoided incidents are statistically different than zero at the level of significance 0.05. Thus, we can conclude that the expected number of avoided incidents is varies between 333 and 458 cases, depending on the approach to calculations, and those differences are statistically significant.

**Table 8:** Expected average number of avoided incidents.

Diabetic incidents	Average RR for treated patients in %/	Avoided incidents for treated patients on average (95% CI)
Any end point related to diabetes	35 (30–38)	141 (121–153)
Death related to diabetes	35 (30–38)	53 (45–57)
All cause mortality	23 (20–26)	47 (41–53)
Fatal and non-fatal myocardial infarction	23 (20–26)	46 (40–52)
Fatal and non-fatal stroke	20 (17–22)	10 (9–11)
Microvascular end points	60 (53–67)	110 (97–123)
Cataract extraction	31 (27–34)	15 (13–16)
Amputation or death from peripheral vascular disease	70 (61–79)	27 (24–31)
Heart failure	26 (23–29)	10 (9–11)
**Total**		458 (398–507)

### Economic Consequences

For a 1-year period based on 2018 prices of observed medicines, the NHIF reimbursed 5.25 million BGN for incretins and SGLT-2i therapy (Table 9). For a 5-year period the expenditures amounted to 26.24 million BGN.

**Table 9:** Reimbursed cost for incretins and SGLT-2i.

	Average reimbursed cost for 2018	Number of patients	Reimbursement expenditures per year per therapeutic group
DPP-4i + MET	41.75	3716	1,861,716
DPP-4i	59.57	947	56,413
GLP-1 RA	172.04	1218	2,514,537
SGLT-2i + MET	47.73	155	88,778
SGLT-2i	83.37	86	86,038
Total per year			5,247,325

National Health Insurance Fund could save between 306 and 510 thousand BGN from avoided incidents for a 5 year therapy.

The costs depend on the way of calculating – per level of HbA1c or an average (Table 10).

**Table 10:** Cost of diabetic incidents.

Diabetes incident	Hospital cost	Yearly ambulatory cost	Difference in the number of incidents	Cost for 10 years therapy	Cost for 10 years therapy of incidents according to level of HbA1c	Cost for 10 years therapy of incidents on average
Any end point related to diabetes	600	427.92	58.9	141	287384.88	687967.2
Death related to diabetes	600	0	46.6	53	27960	31800
All cause mortality	2134	0	40.2	48	85786.8	102432
Fatal and non-fatal myocardial infarction	200	51.22	43.6	47	31051.92	33473.4
Fatal and non-fatal stroke	650.56	43.13	8.4	10	9087.62	10818.6
Microvascular end points	744	36.38	87.6	113	97043.28	125181.4
Cataract extraction	360	10.08	14.4	15	6635.52	6912
Amputation or death from peripheral vascular disease	2050	20.68	21.0	28	47392.8	63190.4
Heart failure	420	112.72	12.0	10	18566.4	15472
Total cost for 10 years ambulatory therapy and only 1 hospital incident for the period			332.7	465	610,909.22	1,077.247

## Discussion

This is the first Bulgarian study based on real life long term data from the diabetes registry for incretins and SGLT-2i therapy. The study presents new, objective information about the therapy, with a new, developing groups of medicine that is incretins and SGLT-2i, estimates the treatment costs and cost of avoided incidents as well as the reduction of RR as a result of the therapy. This is one of the limited numbers of studies on long term therapy with incretins (Freeman and Unger, 2008; Drucker et al., 2010; Ross and Ekoe, 2010). Some of the results concerning this study have been published in a short letter to the editor, whereas the current publication provides more detailed, and analytical information of the observed results (Mitkova et al., 2018). As a whole, the study presents unpublished before and new results about the therapy with a new group of medicines (incretins and SGLT-2i), costs and RR as a result of the therapy.

Our study undoubtedly shows that incretins and SGLT-2i significantly improve type-2 diabetes control by decreasing the HbA1c level for the majority of treated patients and decreasing the RR for diabetes incidents (De Fronzo, 2010; Cernea, 2011; Wang et al., 2016). The results in the current study confirm the significance of treatment. Our study also shows that the control achieved with GLP inhibitors exceeds that achieved with DPP4-inhibitors and that the combination therapy is beneficial in comparison with monotherapy (García-Pérez et al., 2013; Brunton, 2014).

One might argue that the cost of avoided incidents is too small in comparison with the invested reimbursement cost, but this is due to the relatively low cost of health care services in the country (Dimitrov, 2010; Dimitrova et al., 2015). Another reason might also be the fact that the calculations have been done for RR reduction in the case of a 1% decrease in HbA1c level and not on the RR for diabetes control with achieved target HbA1c level that might lead to higher savings. Further studies need to be done in this respect (Brunton, 2014).

Limitations of the study are the different time periods of utilization of different products from the observed therapeutic groups, especially the SGL inhibitors group that did not allow comparison of all products for the whole observed period. Another limitation is that the beneficial effects of incretins and SGLT-2i inhibitors regarding the decrease in hypoglycemic incidents, cardiovascular safety, and wage gain as described in recent publications were not taken into consideration (Xourgia et al., 2017; Mannucci and Monami, 2017). We recognize that the HbA1c level is affected by many factors, not just the medications. It could be influenced by the characteristics of patients, comorbidities, lifestyle, smoking status, and so on. Self-management and adherence toward the non-pharmacological and pharmacological therapies could also have an influence. We have eliminated the other factors that could be considered a limitation of the study with our point of view shaped by the policy of the NHIF toward the prescription of the medicine. As was mentioned, the NHIF require stopping the therapy if there is not a decrease in HbA1c level after a short time period. Therefore, we can consider that the long-term decrease is mostly due to the continuation of the therapy for patients showing a decrease in HbA1c level. This actually provokes our interest to see whether there could be additional long term benefits. Comparison of the data about reduction in diabetic incidents and results of treatment with the different therapeutic groups could be used for a choice of therapeutic options according to patient’s HbA1c levels.

We can recommend further exploration of the long term results and related costs, which will also expand the education of medical professionals for incretins’ clinical benefits (Bakracheva et al., 2002).

## Conclusion

Incretins and SGLT-2i therapy steadily decreases the HbA1c level for the prevailing part of patients to less than 8%. It also increases the number of patients with level of HbA1c below 7%. The total number of patients with poor glycemic control decreased almost 4 times.

The therapy with incretins decreases the risk of developing diabetic incidents to between 333 and 465 cases among 6122 treated patients. Avoided cost for the therapy of diabetes incidents adds to between 305 and 510 thousand BGN.

## Ethics Statement

The Diabetes Register in Bulgaria is open access; the link is http://mail.adiss-bg.com/bitool.html. We have used observational data, which do not required permission. All patients providing information for inclusion in Register were previously informed that the recorded data will be later manipulated according to the Bulgarian Health Act (Ministry of Health, 2016b).

## Author Contributions

All authors participated in drafting of the manuscript and revising it before final submission. In addition to that DT, GA, and VV extracted the data from the registry and provided the patient information. MK and ZM analyzed the medical and social consequences. MM and AS analyzed the economic consequences. KM and ZA performed the statistical analysis. GP designed the study, coordinated the work, and wrote the manuscript, especially the discussion and conclusion section.

## Conflict of Interest Statement

ZA was employed by company Adiss Lab Ltd., Sofia, Bulgaria. All other authors declare no competing interests.

## Abbreviations

DPP-4i: dipeptidyl peptidase-4 inhibitors
GLP-1: glucagon-like peptide 1
HbA1c: glycated hemoglobin
MET: metformin
NHIF: National health insurance fund
RR: relative risk
SGLT-2i: sodium-glucose cotransporter-2 inhibitors.
